# The Acid Sphingomyelinase Inhibitor Amitriptyline Ameliorates TNF-α-Induced Endothelial Dysfunction

**DOI:** 10.1007/s10557-022-07378-0

**Published:** 2022-09-14

**Authors:** Yang Ji, Jing Chen, Lihua Pang, Changnong Chen, Jinhao Ye, Hao Liu, Huanzhen Chen, Songhui Zhang, Shaojun Liu, Benrong Liu, Chuanfang Cheng, Shiming Liu, Yun Zhong

**Affiliations:** 1 Department of Emergency, The Second Affiliated Hospital, Guangzhou Medical University, Guangzhou, 510260 Guangdong China; 2 Department of Cardiology, Guangzhou Institute of Cardiovascular Disease, Guangdong Key Laboratory of Vascular Diseases, State Key Laboratory of Respiratory Disease, the Second Affiliated Hospital, Guangzhou Medical University, Guangzhou, 510260 Guangdong China; 3 Department of Anesthesia, The Second Affiliated Hospital of Guangzhou Medical University, Guangzhou Medical University, Guangzhou, 510260 Guangdong China; 4 Department of Obstetrics, The Second Affiliated Hospital of Guangzhou Medical University, Guangzhou Medical University, Guangzhou, 510260 Guangdong China

**Keywords:** Endothelial dysfunction, Acid sphingomyelinase, Inflammation, Amitriptyline, Atherosclerosis, Mitogen-activated protein kinase

## Abstract

**Purpose:**

Inflammation associated endothelial cell (EC) dysfunction is key to atherosclerotic disease. Recent studies have demonstrated a protective role of amitriptyline in cardiomyocytes induced by hypoxia/reoxygenation. However, the mechanism by which amitriptyline regulates the inflammatory reaction in ECs remains unknown. Thus, the aim of this study was to investigate whether amitriptyline protects against inflammation in TNF-α-treated ECs.

**Methods:**

HUVECs were incubated with amitriptyline (2.5 μM) or TNF-α (20 ng/ml) for 24 h. EdU, tube formation, transwell, DHE fluorescence staining, and monocyte adhesion assays were performed to investigate endothelial function. Thoracic aortas were isolated from mice, and vascular tone was measured with a wire myograph system. The levels of ICAM-1, VCAM-1, MCP-1, phosphorylated MAPK and NF-κB were detected using western blotting.

**Results:**

Amitriptyline increased the phosphorylation of nitric oxide synthase (eNOS) and the release of NO. Amitriptyline significantly inhibited TNF-α-induced increases in ASMase activity and the release of ceramide and downregulated TNF-α-induced expression of proinflammatory proteins, including ICAM-1, VCAM-1, and MCP-1 in ECs, as well as the secretion of sICAM-1 and sVCAM-1. TNF-α treatment obviously increased monocyte adhesion and ROS production and impaired HUVEC proliferation, migration and tube formation, while amitriptyline rescued proliferation, migration, and tube formation and decreased monocyte adhesion and ROS production. Additionally, we demonstrated that amitriptyline suppressed TNF-α-induced MAPK phosphorylation as well as the activity of NF-κB in HUVECs. The results showed that the relaxation response of aortic rings to acetylcholine in the WT-TNF-α group was much lower than that in the WT group, and the sensitivity of aortic rings to acetylcholine in the WT-TNF-α group and WT-AMI-TNF-α group was significantly higher than that in the WT-TNF-α group.

**Conclusion:**

These results suggest that amitriptyline reduces endothelial inflammation, consequently improving vascular endothelial function. Thus, the identification of amitriptyline as a potential strategy to improve endothelial function is important for preventing vascular diseases.

**Supplementary Information:**

The online version contains supplementary material available at 10.1007/s10557-022-07378-0.

## Introduction

Atherosclerosis (AS) is known as a chronic inflammatory disease of the endothelium, and the pathogenic mechanism of atherosclerosis includes the expression of cytokines/chemokines, activation of proinflammatory signaling pathways and increased oxidative stress [1–3]. Endothelial cells, as the innermost layer in all vessels, are crucial in regulating tissue homeostasis by controlling the extravasation of circulating cells into tissues. This process is achieved by endothelial cell-driven alteration of cytokine, chemokine, and adhesion molecule production and regulation of the movement of immune cells into the site of injury [4, 5]. ECs targeted by the proinflammatory TNF-α are regulated in multiple functions, including adhesion, thrombosis, and the inflammatory response [6–11]. Endothelial dysfunction promotes the adhesion and migration of macrophages, which are widely recognized as critical to the initiation and progression of atherosclerotic lesions [12, 13].

Sphingolipids are key structural cell membrane lipid compounds. Ceramide (CER) is generated from sphingomyelin (SM), which is hydrolyzed by sphingomyelinase (SMase), triggering a cascade of bioactive lipids. SMase activation occurs in different types of cardiovascular cells, such as cardiomyocytes (CMs), ECs, and vascular smooth muscle cells (VSMCs) to mediate cell proliferation, cell death, cardiac fibrosis [14], and contraction of cardiomyocytes [15, 16].

SMase includes three isoforms based on their optimum pH (acid, neutral, and alkaline) [17]. The direct metabolites of SMase are recognized as second messengers in various specific biological activities such as oxidized lipoprotein (ox-LDL)-induced cell proliferation [18, 19] and TNF-α [20, 21] induced expression of adhesion molecules. Acid sphingomyelinase (ASMase) is expressed by most human tissues, and is induced by proinflammatory cytokines, lipopolysaccharide (LPS), and cytotoxic agents [22–24]. Blocking the production of CER by suppressing ASMase is a potential therapeutic strategy against cardiovascular diseases [25, 26]. Numerous studies have shown that different types of SMase inhibitors, as well as SMase deficiency, can relieve atherosclerosis symptoms [23, 27]. Inhibition of ASMase has been used as a treatment against ischemia reperfusion injury and atherosclerosis [23, 28]. Therefore, the search for safe and effective inhibitors could provide a possible strategy for the prevention of vascular diseases.

Amitriptyline (AMI) is a secondary amine tricyclic antidepressant that mainly inhibits noradrenaline uptake and lysosomal acid sphingomyelinase (ASMase) [29]. Lu et al. demonstrated that AMI is an effective inhibitor of nonalcoholic steatohepatitis (NASH) in LDL receptor-deficient (LDLR^−/−)^ mice with type 2 diabetes mellitus (T2DM) [30]. However, the underlying mechanism by which AMI attenuates atherosclerosis remains unclear.

In this study, we aimed to determine whether AMI or ASMase knockout could increase the phosphorylation of endothelial nitric oxide synthase (eNOS) and the activity of ECs in vivo and in vitro, and inhibit TNF-α induced reactive oxygen species (ROS) production, inflammation, and cell adhesion. We also explored the underlying mechanisms.

## Materials and Methods

### Animals

The animal handing and experimental procedures were in accordance with the NIH regulations for the care and use of animals in research, and were approved by the Animal Ethics and the Use Committee of the Second Affiliated Hospital of Guangzhou Medical University. The 8 to 12-week-old wild-type (WT) C57BL/6J male mice were purchased from the Guangdong Medical Laboratory Animal Center (Guangzhou, China). ASMase knockout (ASMase^−/−)^ mice on a C57BL/6 background were obtained from Cyagen Biosciences Inc (supplementary Fig. 1). As previously described, all the mice were housed in a 12 h dark/light cycle with unlimited access to food and water. After an initial adaptation period, the mice were randomly assigned to five groups: 1. WT mice (control group), 2. WT mice with TNF-α treatment (WT-TNF-α group), 3. WT mice with TNF-α and amitriptyline treatment (WT-AMI-TNF-αgroup), 4. ASMase^−/−^ mice (ASMase^−/−^ group), and 5. ASMase^−/−^ mice with TNF-α treatment (ASMase^−/−^-TNF-α group) (*n* = 5 per group). The mice in group 3 received 5.0 mg/kg of amitriptyline by intragastric administration for 30 days. During the last four days, the mice in groups 2, 3, and 5 were intraperitoneally injected with recombinant mouse TNF-α protein (50349 Sino Biological, Beijing, China) at a dose of 30 μg/kg body weight for 4 consecutive days. Saline was used as a negative control during the same period. After the experiment, all mice were fasted for 12 h and anaesthetized by inhalation of 2.0% isoflurane followed by cervical dislocation.

### Vascular Tone Measurement

The thoracic aortic rings (2–3 mm in length) were removed immediately after the mice were killed and the surrounding tissues were gently removed and immersed in oxygenated modified physiological saline solution (PSS) containing (in g/L) NaCl, 7.598; MgSO_4_, 0.29; KCl, 0.35; KH_2_PO_4_, 0.26; NaHCO_3_, 1.25; glucose, 1.0; CaCl_2_, 0.178; and Na_2_-EDTA, 0.01 (pH 7.4). The rings were then cut and mounted onto a 4-channel wire myograph system (610M; DMT, Aarhus. Denmark) in a 5-mL organ chamber filled with PSS and gassed with 95% O_2_ and 5% CO_2_ at 37°C. The artery ring was stretched to a resting tension of 9 mN and acclimated for 30 mins; the PSS was replaced every 15 min. The solution was replaced twice with 60 mmol/L of high potassium physiological saline solution (KPSS) containing (in g/L) NaCl, 4.37; MgSO_4_, 0.29; CaCl_2_, 0.178; KCl, 4.47; KH_2_PO_4_, 0.26; NaHCO_3_, 1.25; glucose, 1.0; and Na_2_-EDTA, 0.01 (pH 7.4) to test for maximal contraction. After adequate washing with PSS and equilibration, the aortic tissues were examined for endothelium-dependent relaxation under cumulative concentrations of acetylcholine (10^−10^ to 10^−6^ mol/L) and endothelium-independent relaxation under cumulative concentrations of sodium nitroprusside (SNP, 10^−11^ to 10^−7^ mol/L) after submaximal contraction with phenylephrine (0.1 μmol/L). For analysis of the potential roles of NOS in changes in relaxation, the NOS blocker L-NAME (10 μmol/L) was added to the bath for 30 min before the application of Ach, and then Ach-induced vasodilatation was re-examined.

### Cell Culture and Treatment

HUVECs were obtained from Cell Applications Inc. (Supplementary Fig. 2). Human THP-1 monocytes were grown in suspension at a cell density between 10^5^ and 10^6^ cells/mL in RPMI 1640 with 10% FBS and 1% penicillin/streptomycin. HUVECs were cultured in EBM-2 as described previously [31]. After HUVECs reached 70–80% confluence, they were starved in FBS-free EBM-2 for 12 h and then preincubated with AMI (0.625–5 μM) for 1 h before being incubated with TNF-α (20 ng/ml) for 24 h. AMI was also present in the treatment medium alongside TNF-α.

### Acid Sphingomyelinase Activity Assay

An ASMase assay kit (ab190554, Abcam) was used to assay ASMase activity in HUVECs. Briefly, the cells were lysed with mammalian cell lysis buffer (ab179835, Abcam) and then the samples were reacted with ASMase assay reagents according to the protocol recommended by the manufacturer. After the incubation, fluorescence from each sample was detected at Ex/Em=540/590 nm using a microplate reader.

### Cell Viability Assay

The viability of HUVECs was determined using the Cell Counting Kit-8 (CCK-8, Dojindo, CK04-11). HUVECs were seeded in 96-well plates and allowed to reach 70% confluence. After treatment with AMI for 24 h, the cells were incubated in 100 μL of fresh serum-free medium containing 10 μL CCK-8 reactive solution for 3 h. The absorbance of the supernatant was detected at 450 nm according to the manufacturer’s instructions.

### Western Blotting

The protein levels of ICAM-1, VCAM-1, MCP-1, phospho-eNOS, phospho-transcription factor nuclear factor-κB (NF-κB), phospho-p44/42 mitogen-activated protein kinase (MAPK), phospho-SAPK/JNK MAPK, and phospho-P38 MAPK were analyzed using western blotting. As described previously [32], cells were washed with PBS twice and then lysed in 65 μL of ice-cold RIPA lysis buffer (Beyotime, Shanghai, China). The protein concentration was measured using the BCA Protein Assay Kit (Thermo Fisher, USA). Total protein (10–20*μg*) was separated by SDS-PAGE and transferred to PVDF membranes (0.45*μm, Millipore*, USA). Then, the membranes were blocked with 5% nonfat milk in Tris-buffered saline Tween (TBST) at room temperature for 1 h and incubated overnight at 4 °C with primary antibodies against VCAM-1 (1:1000, Cell Signaling Technology, USA, 13662), ICAM-1 (1:1000, Cell Signaling Technology, USA, 67836), MCP-1 (1:1000, Cell Signaling Technology, USA, 39091), phospho-eNOS (Ser1177, 1:1000, Cell Signaling Technology, USA, 9570), phospho-p44/42 (Thr202/Tyr204,1:1000, Cell Signaling Technology, USA, 9101), phospho-SAPK/JNK (Thr183/Tyr185,1:1000, Cell Signaling Technology, USA, 4668), phospho-P38 (Thr180/Tyr182,1:1000, Cell Signaling Technology, USA, 4511), phospho-NF-κB (Ser536,1:1000, Cell Signaling Technology, USA, 3033), ASMase (1:1000, ABclonal, China, A6743), β-actin (1:1000, Cell Signaling Technology, USA, 4970), and GAPDH (1:1000, Cell Signaling Technology, USA, 3261). After incubation with the secondary anti-rabbit antibody (1:5000, Jackson, USA, 144208), immune complexes were detected with an ECL western blotting substrate (Affinity, USA, KF005). Densitometry was performed using ImageJ (1.52v, USA) software.

### Immunofluorescence

The expression of ICAM-1 was analyzed by immunofluorescence, which was performed as previously described [33]. In brief, vascular tissues from mice were fixed with 4% paraformaldehyde, fixed in paraffin, and sectioned into slices. Then, the tissues were blocked with BSA. The slices were incubated with the primary antibody overnight at 4 °C, and then incubated with the secondary antibody for 60 min at room temperature. Then, the slices were incubated with DAPI for 10 min at room temperature. Fluorescence images were captured by inverted fluorescence microscopy. The primary antibodies against ICAM-1 (cat.gb11306) and CD31 (cat.gb12063) were purchased from Wuhan Servicebio Technology Co., Ltd (Wuhan, China).

### Nitric Oxide (NO) Detection

According to a previously described method [34], the NO level in the supernatant was measured by the nitrate reductase method according to the instructions of the NO determination kit (No. A012, Nanjing Jiancheng, China). Absorbance was detected using a spectrophotometer at a wavelength of 550 nm.

### Proliferation Assay

The effects of AMI on cell proliferation were determined by EdU staining (Riobio, C10310-3). Cells (5×10^3^/well, five replicates per group) were seeded in 96-well plates and incubated with 10 nM EdU for 4 h. Cells were washed, fixed, and stained according to the manufacturer’s instructions. Cells were observed and counted using a fluorescence microscope.

### Adhesion of Monocytes Assay

The measurement of adhesive monocytes was performed according to a previously described method [35]. HUVECs were cultured in 24-well glass chamber slides. THP-1 cells were labeled with 5 μg/ml calcein-AM in EBM-2 medium containing 5% FBS. Calcein-AM-labeled THP-1 cells (4.0×10^5^ cells/mL) were seeded on AMI-treated and TNF-α-treated ECs and coincubated for 45 min. Images were obtained using a fluorescence microscope.

### Enzyme-Linked Immunosorbent Assay (ELISA)

ELISAs were performed on the cell supernatant to determine the levels of ceramide (11268, Ruxinbio Quanzhou, China), sICAM-1 (1113542, Dakota, China), and sVCAM-1 (1114062, Dakota, China), according to the manufacturer’s instructions.

### Transwell Assay

The EC migration assay was performed using Costar Transwell cell culture chambers (354234, Corning, USA) according to the manufacturer’s instructions. HUVECs were preconditioned and seeded (4×10^4^ cells per well) in the top chambers with 8.0-μm pore polycarbonate membrane inserts. Cells were then incubated in serum-free medium in the upper chamber, and medium with 3% FBS was added to the bottom chambers. Cells were incubated for 16 h, and cells in the lower layer were washed, fixed, stained with crystal violet solution (Sigma-Aldrich), and observed under a microscope.

### Tube Formation

Matrigel (356234, Corning) was used to coat the 96-well plates and allowed to solidify (37°C, 45 min), prior to seeding with HUVECs. Cells (3×10^5^/ml) were incubated at 37 °C for 6 h in serum free EBM-2 medium. Images of the tube network were obtained using a microscope, and the total length of the tubes were analyzed using Image J software (Bethesda, MD, USA, USA).

### Detection of ROS Generation by DHE Fluorescence Staining

The ECs were incubated with dihydroethidium (DHE, 20 μM) in EBM-2 for 45 min, followed by three washes with PBS. Quantification of fluorescent images was performed with ImageJ software (Bethesda, MD, USA, USA).

### Statistical Analysis

Data are presented as the mean ± standard error of mean (SEM). Statistical analysis was performed using SPSS 21.0 software (IBM Corporation, Armonk, NY, USA). Student’s *t* test was employed to compare data between two groups or multigroup comparisons were performed through one-way analysis of variance, followed by a post hoc Tukey test. A *P* < 0.05 denoted a statistically significant difference.

## Results

### Amitriptyline Inhibited TNF-α-Induced ASMase Activation

**Fig. 1:**
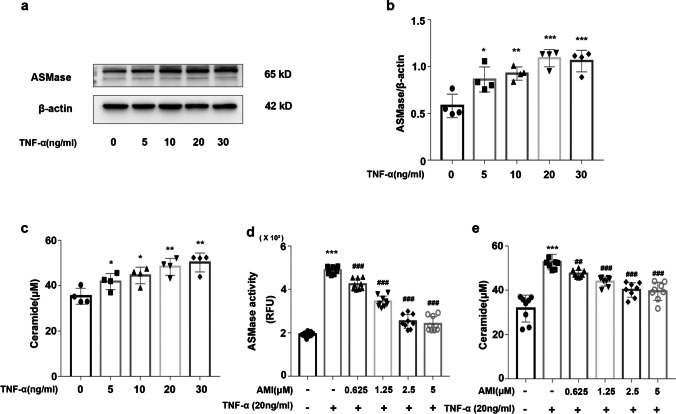
Amitriptyline inhibited TNF-α-induced ASMase activation (**a**) Western blotting data showing effects of TNF-α on expression levels of ASMase in HUVECs. (**b**) Statistical results of relative protein expression. (**c**) The effect of TNF-α on ceramide levels in the supernatant. (**d**) Activity of ASMase in HUVECs. (**e**) The effect of AMI (0–5 μM) on ceramide levels in the supernatant of TNF-α-activated HUVECs as measured by ELISA Data are represented as the mean±SEM (*n* = 3–6). All the experiments were performed independently three times. *P* < 0.05, **P < 0.01, ***P< 0.001 vs control; ^#^*P* < 0.05, ^##^*P* < 0.01, ^###^*P* < 0.001 vs TNF-α

To explore the effect of TNF-α and amitriptyline on ASMase, we assessed the protein expression and activity of ASMase and the release of ceramide. The western blot and ELISA results showed that in the EC treated with TNF-α (5 ng/ml to 30 ng/ml), the protein expression of ASMase and the release of ceramide were increased in a dose-dependent manner (Fig. 1a-c). The CCK-8 results showed that EC viability was not significantly different after treatment with TNF-α (5 ng/ml to 30 ng/ml) (Supplementary Fig. 3a). ASMase activity assays and ELISAs were performed to test the inhibitory effect of amitriptyline on the ASMase activity of ECs. The results showed that the increased ASMase activity and ceramide release were inhibited by pretreatment with AMI at 0.625, 1.25, 2.5, and 5 μM (Fig. 1d-e). Thus, amitriptyline was regarded as an active inhibitor of ASMase.

### Amitriptyline Enhanced the Function of Endothelial Cells

**Fig. 2:**
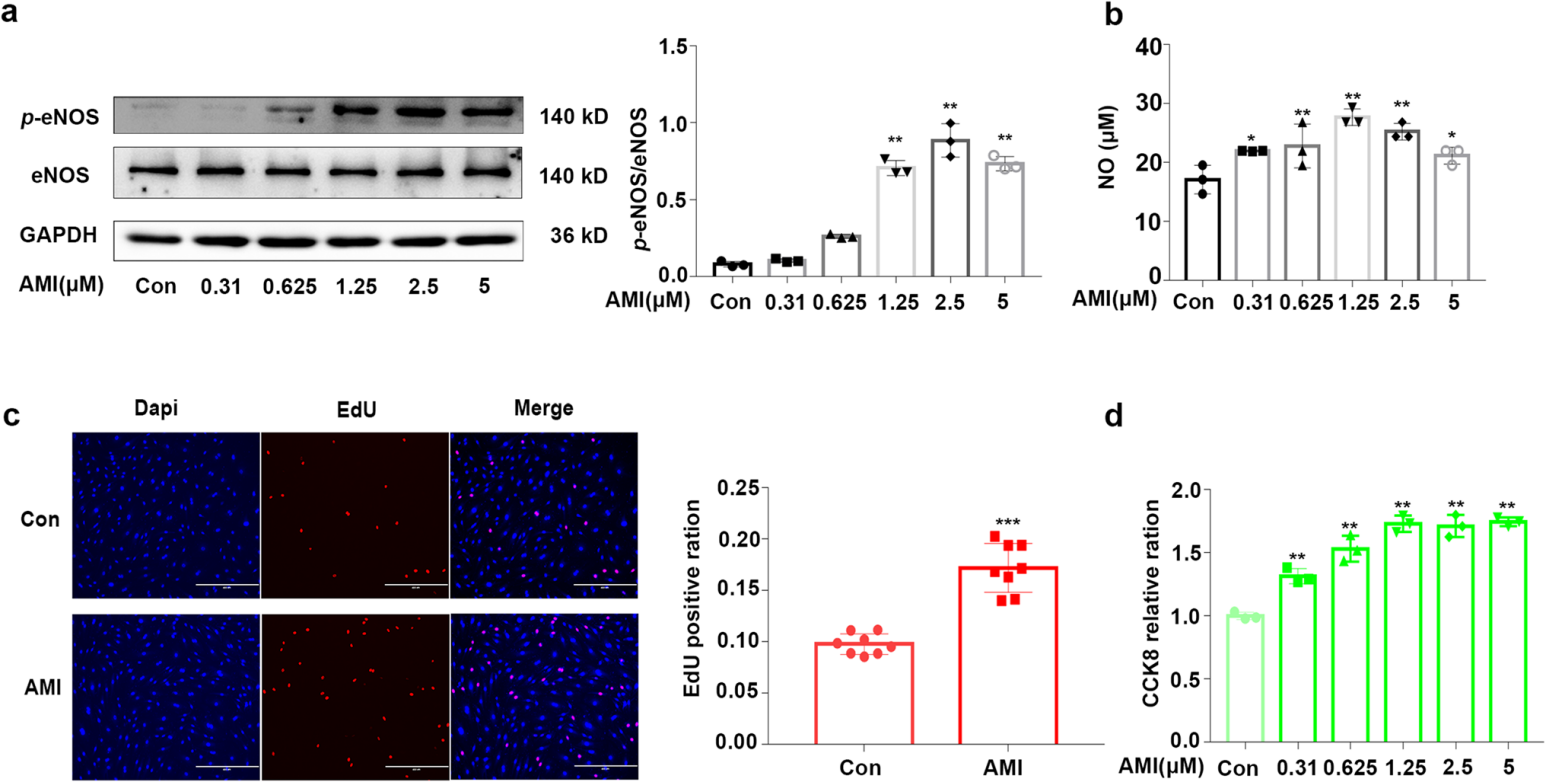
Amitriptyline enhanced the function of endothelial cells (**a**) Representative western blotting images and summarized data showing the effect of AMI (0–5 μM, 24 hours) on phosphorylated eNOS. (**b**) NO release from endothelial cells induced by AMI (0–5 μM, 24 h). (**c**) The effect of AMI on HUVECs proliferation. (**d**) Effects of AMI (0–5 μM, 24 h) on the viability of HUVECs. Bar: 400 μm in (**c**). Data are represented as the mean±SEM; All the experiments were performed independently three times. *P < 0.05, **P < 0.01, ***P < 0.001 vs control

NO bioavailability plays a vital role in the function of endothelial cells. To measure AMI-induced NO bioavailability in endothelial cells, we performed western blotting to identify the phosphorylation of eNOS. The results showed that the phosphorylation degree of eNOS increased following AMI treatment in a dose-dependent manner (Fig. 2a). The release of NO from endothelial cells was also measured via nitrate reductase levels. In accordance with the western blotting results, AMI also elevated the amount of nitrite oxide released into the medium of HUVECs (Fig. 2b). To determine the effect of AMI on ECs, we performed multiple functional assays, including viability and proliferation assays. As shown in Fig. 2c and d, the HUVECs incubated with AMI showed a marked increase in proliferation (75% with EdU staining) and cell viability (69% in the CCK-8 assay) compared to the control cells. Therefore, these results indicated that AMI increased EC proliferation, viability, and the release of NO.

### Amitriptyline Inhibited ICAM-1 and VCAM-1 Expression and Monocyte Adhesion in TNF-α-Treated Endothelial Cells

**Fig. 3:**
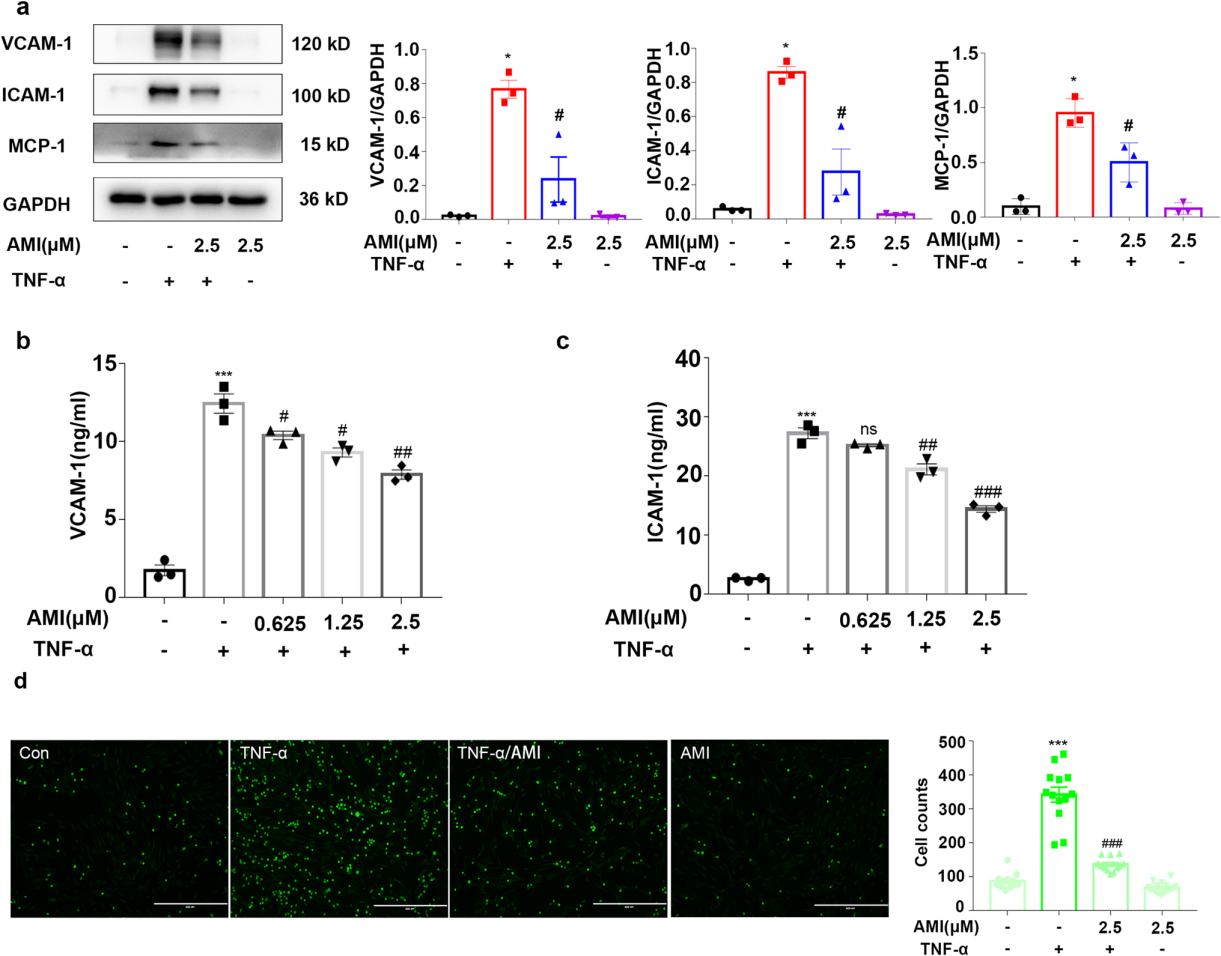
Amitriptyline inhibited ICAM-1 and VCAM-1 expression and monocyte adhesion in TNF-α-induced endothelial cells (**a**) Western blotting data showing effects of AMI on expression levels of VCAM-1, ICAM-1, and MCP-1 in TNF-α stimulated HUVECs. (**b**, **c**) The effect of AMI (0, 0.625, 1.25, 2.5 μM) on sVCAM-1 levels in the supernatant (**b**), sICAM-1 (**c**) in TNF-α-activated HUVEC as measured by ELISA. (**d**) The effect of AMI on THP-1 monocyte adhesion to TNF-α-activated HUVECs. Bar: 400 μm in (**d**). The concentrate of TNF-α was 20 ng/ml. Data are represented as the mean±SEM (*n* = 3–6). All the experiments were performed independently three times. *P < 0.05, **P < 0.01, ***P< 0.001 vs control; ^#^*P* < 0.05, ^##^*P* < 0.01, ^###^*P* < 0.001 vs TNF-α

Monocyte adherence is one of the original events in atherosclerosis. To determine whether AMI regulates the adhesion of monocytes, we first explored the effect of AMI on adhesion molecule expression induced by TNF-α in HUVECs by western blotting. As shown in Fig. 3a, AMI alone did not cause any significant effects. However, the protein expression of adhesion molecules was significantly increased after the administration of TNF-α (20 ng/ml) compared with that of the control group. AMI (2.5 μM) decreased the enhanced expression of ICAM-1, VCAM-1, and MCP-1. ELISAs showed similar results (Fig. 3b, c).

To further identify the role of AMI in TNF-α-induced monocyte recruitment to the vascular endothelium, we performed cell adhesion assays using THP-1 monocytes and HUVECs (Fig. 3d). A small number of monocytes adhered to HUVECs in the absence of TNF-α. However, after treatment with TNF-α for 24 h, the number of adherent THP-1 cells increased approximately 300%. In addition, pretreatment of the cells with AMI inhibited TNF-α-induced monocyte adhesion to endothelial cells. No significant difference in THP-1 cell count was observed between the AMI alone and control groups. These results indicated that AMI blocked monocyte migration in response to inflammatory mediators by inhibiting the expression of endothelial adhesion molecules.

### Amitriptyline Prevented TNF-α-Mediated EC Dysfunction

**Fig. 4:**
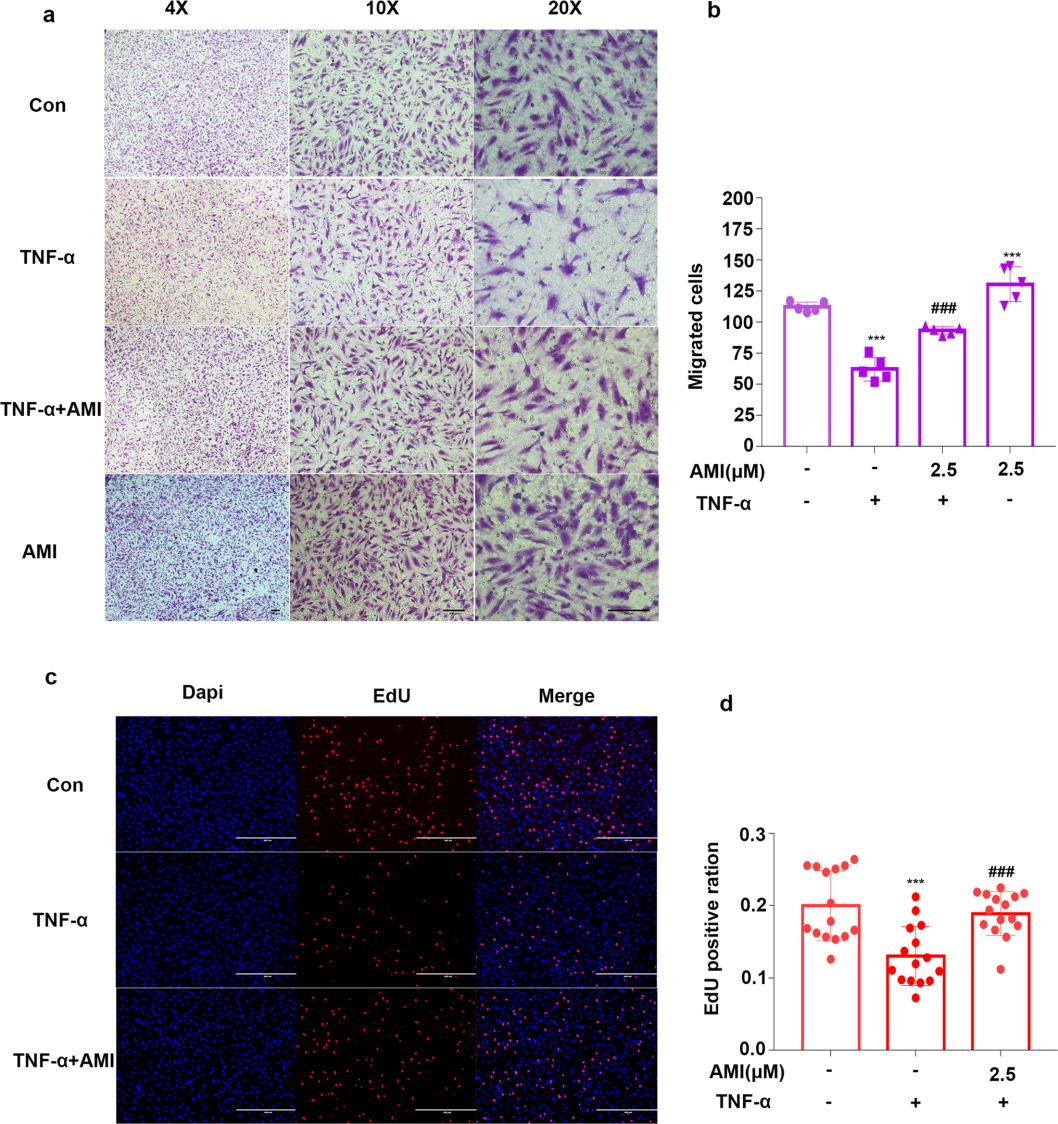
Amitriptyline prevented TNF-α-mediated ECs dysfunction (**a**) AMI attenuated TNF-α effects on HUVECs in the transwell (8 μm) assay. (**b**) Quantitative analysis of cell migration in (**a**). (**c**) AMI attenuated the effect of TNF-α in HUVECs as measured by EdU staining. (**d**) Quantitative analysis of the proliferation rates in (**c**) (*n* = 5). Bar: 200 μm in (**a**), 400 μm in (**c**). Data are represented as the mean±SEM (*n* = 5). The concentrate of TNF-α was 20 ng/ml. All the experiments were performed independently three times. *P < 0.05, **P < 0.01, ***P < 0.001 vs control; ^#^*P* < 0.05, ^##^*P* < 0.01, ^###^*P* < 0.001 vs TNF-α

**Fig. 5:**
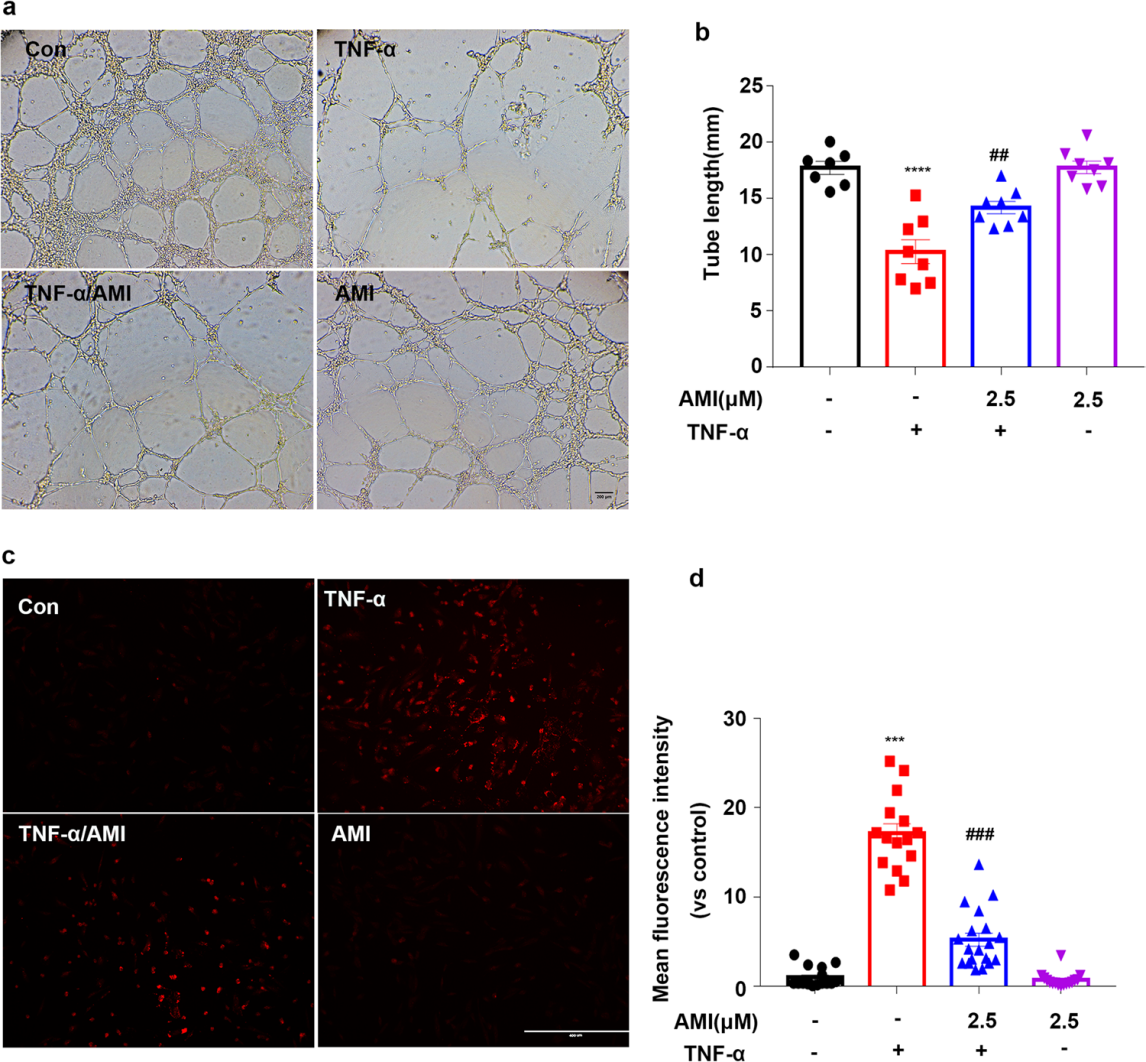
Treatment with AMI improved tube formation and decreased oxidase-dependent ROS generation in HUVECs mediated by TNF-α (**a**) AMI alleviated the inhibitory role of TNF-α on angiogenesis in HUVECs. (**b**) Quantification of total branch. (**c**, **d**) Representative fluorescent images of DHE staining (**c**). Summary data (**d**) showing the roles of AMI in the production of ROS in HUVECs treated with TNF-α. Bar: 200 μm in (**a**), 400 μm in (**c**). The concentrate of TNF-α was 20 ng/ml. Data are represented as the mean±SEM (*n* = 5). All the experiments were performed independently three times. *P < 0.05, **P < 0.01, ***P < 0.001 vs control; ^#^*P* < 0.05, ^##^*P* < 0.01, ^###^*P* < 0.001 vs TNF-α

We then investigated the effect of AMI on the function of ECs induced by TNF-α. TNF-α is an important risk factor for atherosclerosis, as it impairs endothelial cell function [35–37]. First, the effect of AMI on angiogenic characteristics, including proliferation, migration and tube formation, of EC were examined. A transwell coculture system (8 μm) was used to detect the migration of ECs. We found that TNF-α stimuli decreased the migration of HUVECs, while pretreatment with AMI clearly increased migration (Fig. 4a, b). As shown in Fig. 4c and d, the HUVECs treated with TNF-α exhibited a lower EdU staining intensity than that of the control group, indicating inhibition of cell proliferation. However, the HUVECs preincubated with AMI showed markedly improved proliferation compared with those preincubated with TNF-α. We next determined whether AMI improved blood vessel development using a Matrigel assay in cultured HUVECs, TNF-α strongly suppressed tube formation in normal HUVECs, whereas pretreatment with AMI restored angiogenesis (Fig. 5a, b). Therefore, these data indicated that AMI prevented TNF-α-induced endothelial dysfunction.

### Amitriptyline Prevented TNF-α-Stimulated ROS Production

Oxidative stress is a key factor contributing to the pathogenesis of atherosclerosis [38–40]. DHE staining showed that after incubation with TNF-α for 24 h, the level of ROS was significantly augmented (Fig. 5c). However, AMI pretreatment significantly mitigated TNF-α-induced ROS generation in HUVECs. These data suggested that AMI prevented TNF-α-stimulated ROS production.

### Amitriptyline Reversed the TNF-α-Induced Increase in MAPK Activity

**Fig. 6:**
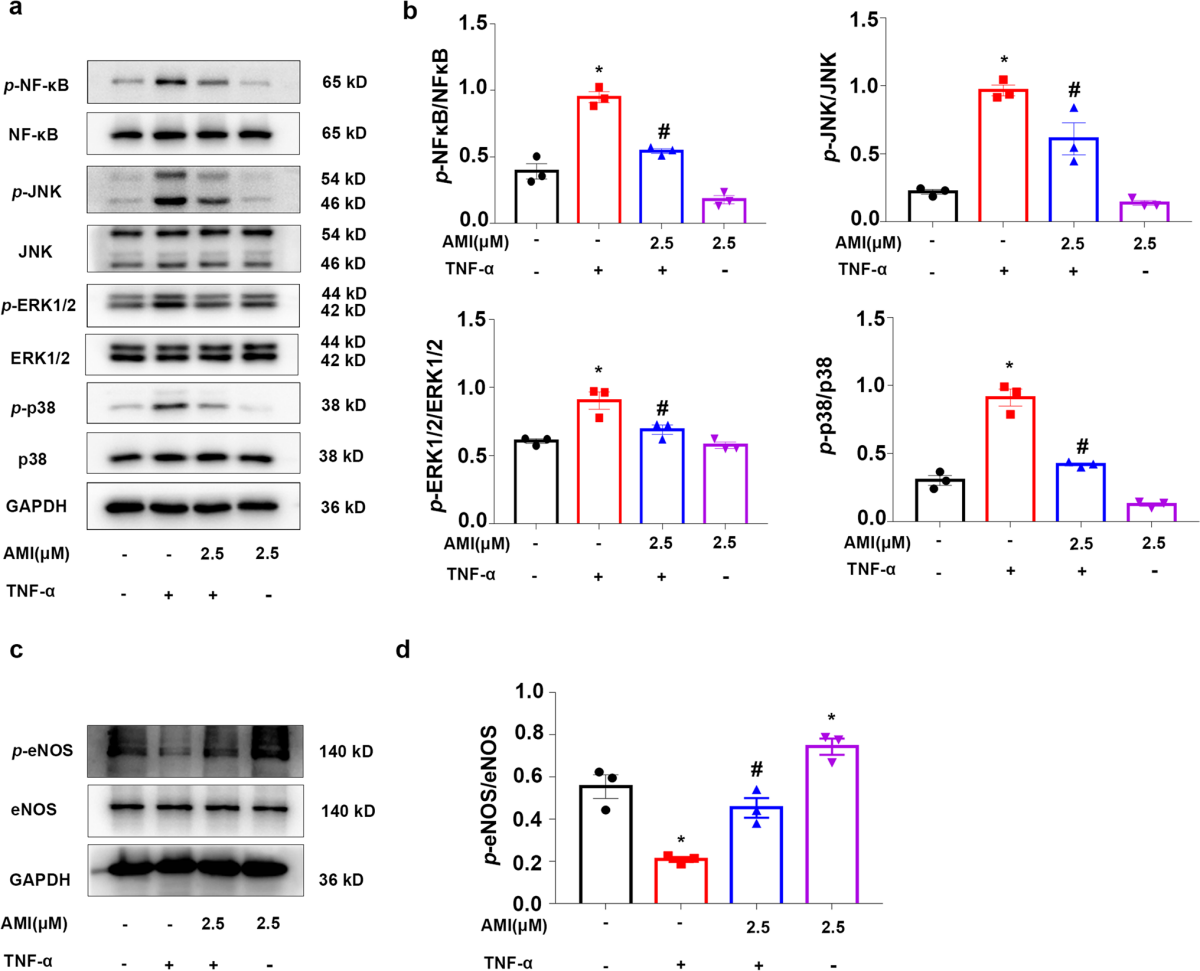
Amitriptyline reversed TNF-α-induced increased MAPK and NF-κ B activity Western blotting and (**a**) statistical analysis (**b**) of expression of phosphor-NF-κ B, phosphor-ERK, phosphor-P38, and phosphor-JNK in TNF-α treated HUVEC. Data are represented as the mean±SEM (*n* = 3). (**c**–**d**) Western blotting data showing effects of AMI on expression levels of *p*-eNOS in TNF-α stimulated HUVEC. The concentrate of TNF-α was 20 ng/ml. All the experiments were performed independently three times. *P < 0.05, **P < 0.01, ***P < 0.001 vs control; ^#^*P* < 0.05, ^##^*P* < 0.01, ^###^*P* < 0.001 vs TNF-α

MAPK signaling is activated by TNF-α binding to the receptor [41]. Therefore, we investigated whether AMI inhibits TNF-α induced proinflammatory effects in ECs through the MAPK signal transduction pathway. Western blotting results showed that TNF-α significantly enhanced the phosphorylation of NF-κB, p38 MAPK, p44/42 MAPK, and JNK MAPK following various treatments. However, this increased phosphorylation was significantly attenuated by treatment with AMI (Fig. 6a, b). No difference in MAPK activation was observed between the AMI alone and control groups. The phosphorylation degree of eNOS decreased approxiamately 60% after incubation with TNF-α; similarly, the decrease in TNF-α-induced ECs could be rescued by AMI (Fig. 6c, d). Therefore, the results indicated that AMI treatment attenuated MAPK and NF-κB activation in TNF-α-treated ECs.

### AMI and Knockout of ASMase Improved Vascular Function in Mice Intraperitoneally Injected with TNF-α

**Fig. 7:**
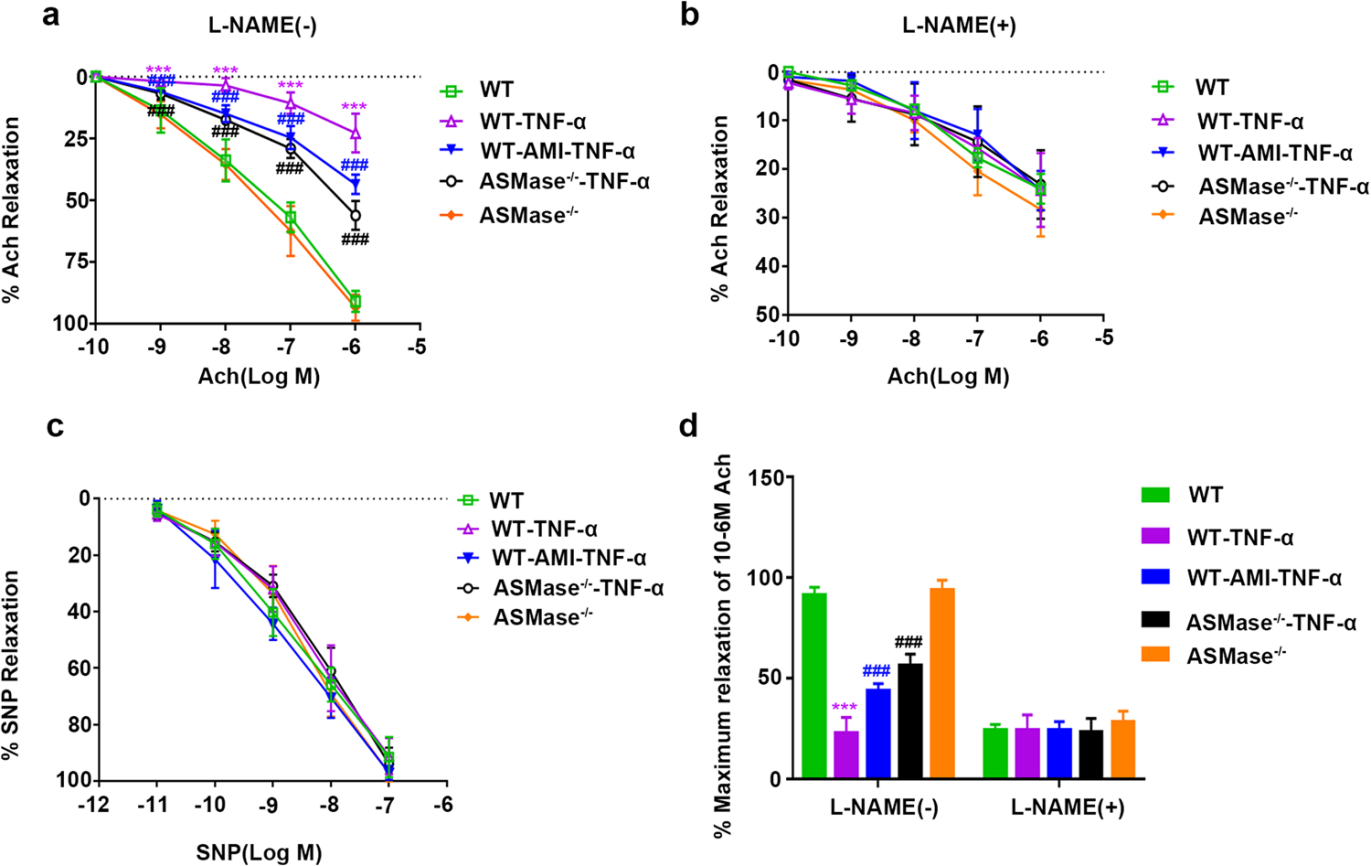
The role of ASMase in arterial relaxation after intraperitoneal (i.p.) injection of recombinant mouse TNF-α (**a**, **b**) Cumulative concentration-dependent Ach-induced relaxation of mouse aortic vessels in the absence (**a**) and presence of L-NAME (**b**). (**c**) The relaxation concentration-response curve to SNP was attained in mouse aortic vessels. (**d**) Maximum relaxation of aortic vessels with or without L-NAME in response to 10^-6^ M Ach. Data are represented as the mean±SEM (*n* = 5). All the experiments were performed independently three times. *P < 0.05, **P < 0.01, ***P < 0.001 vs WT; ^#^*P* < 0.05, ^##^*P* < 0.01, ^###^*P* < 0.001 vs WT- TNF-α

The changes in ceramide in mouse plasma were measured by ELISAs. The results showed that TNF-α (30 μg/kg BW) increased ceramide in mouse plasma, and amitriptyline (5mg/kg) and knockout of ASMase reduced ceramide in mouse plasma (Supplementary Fig. 3b). Endothelium-dependent relaxation is associated with vascular function. To explore the role of ASMase and AMI in arterial relaxation after intraperitoneal injection of recombinant mouse TNF-α, we measured the vasodilative response of isolated aortic segments to endogenic and exogenic NO. PE-precontracted aortas from the ASMase^−/−^ and WT mice were analyzed. As shown in Fig. 7a, the diastolic response to acetylcholine of the TNF-α group was far weaker than that of the WT group, indicating that endothelium-dependent diastolic function was impaired under TNF-α. In the ASMase^−/−^-TNF-α group and the WT-AMI-TNF-α group, the aortic rings were much more sensitive to acetylcholine than the those in the TNF-α group. Treatment with the NO inhibitor L-NAME (10 μM) suppressed acetylcholine-induced endothelium-mediated vasodilation, and there was no significant difference in vasodilation among the different groups of mice (Fig. 7b). However, these results were not found under SNP-induced endothelium-independent vasodilation, which represents the relaxation function of smooth muscle (Fig. 7c). Thus, the results showed that ASMase impaired NO release to regulate endothelium-mediated vasodilation and vascular tone. AMI and knockout of ASMase enhanced NO bioavailability and secondary vasodilation after treatment with TNF-α.

### AMI and Knockout of ASMase Decreased the Expression of Vascular ICAM-1 in Mice Intraperitoneally Injected with TNF-α

**Fig. 8:**
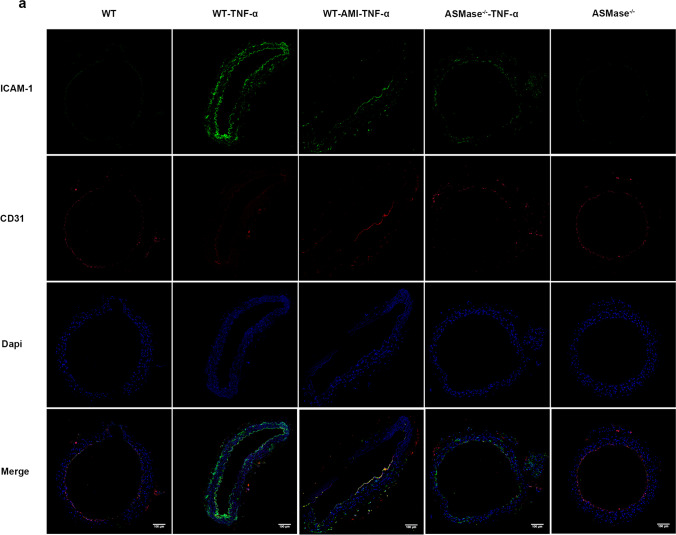
Expression of ICAM-1 and CD31 in the vessels of the mice. Immunofluorescence of ICAM-1 and CD31 in the vascular endothelium. The cells that expressed sufficient ICAM-1 were stained green, and the cells that expressed sufficient CD31 were stained red.n=5

To further determine the role of AMI in inhibiting vascular inflammation in vivo, we detected CD31, a specific marker of vascular ECs, and ICAM-1 by immunofluorescence. The results showed that the expression of ICAM-1 (mainly located in the endothelium) was obviously upregulated in the WT-TNF-α group, while AMI and knockout of ASMase ameliorated decreased the expression of vascular ICAM-1 in mice intraperitoneally injected with TNF-α (Fig. 8).

## Discussion

**Fig. 9:**
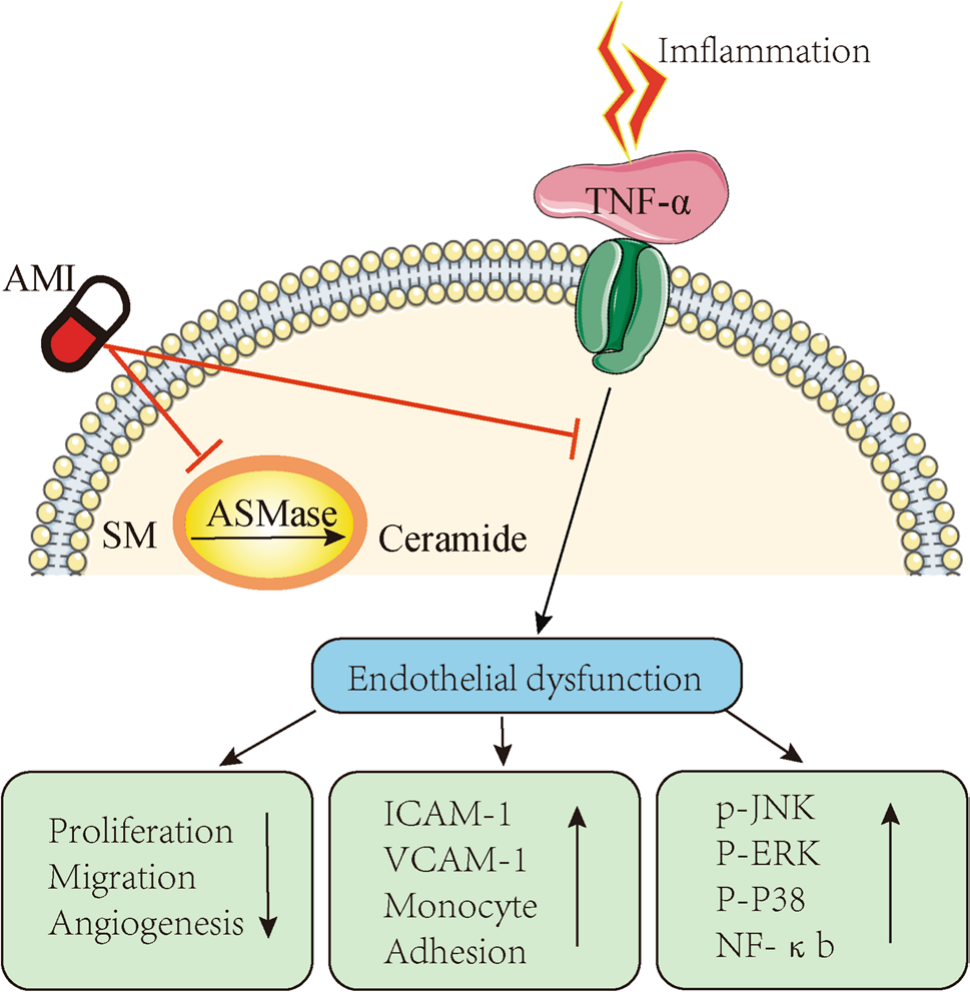
Diagram of the signaling cascades involved in the effect of AMI on TNF-α signaling pathways in ECs. AMI, amitriptyline; SM, sphingomyelin; ASMase, acid sphingomyelinase

This study revealed that AMI, an inhibitor of lysosomal acid sphingomyelinase, elevated eNOS phosphorylation and endogenous NO production in HUVECs. Preincubation with AMI prevented TNF-α-induced endothelial dysfunction. AMI also decreased the activation of MAPK signaling and inhibited the activity of ASMase and the release of ceramide after treatment with TNF-α, in turn reducing the expression levels of adhesion molecules and ROS. After treatment with TNF-α, endothelial-dependent relaxation of the aorta in the ASMase^–/–^ mice and the mice treated with amitriptyline was increased compared with those of their wild-type counterparts (Fig. 9).

EC dysfunction, including the imbalance of vasodilation and vasoconstriction, increased ROS generation, proinflammatory cytokine production, and insufficient NO bioavailability, contributes to atherosclerosis [34, 42]. Additionally, mainly located in the lysosomal sections, ASMase hydrolyzes SM to phosphocholine and CER, which is linked with atherosclerosis [43]. In the present study, we observed that TNF-α significantly increased the expression of ASMase and the release of ceramide in ECs, demonstrating a vital role of ASMase in endothelial inflammation.

AMI has been widely used in clinical practice as an antidepressive therapy, however, its therapeutic action may not merely benefit people with depression [44]. AMI can antagonize many receptors, such as the serotonin receptor. Furthermore, AMI is a functional inhibitor of ASMase [29]. Therefore, it is likely that AMI may regulate cellular function through multiple biological pathways [30]. Dai et al. revealed that AMI attenuated cardiomyocyte apoptosis induced by hypoxia/reoxygenation [45]. In this study, we discovered that AMI significantly inhibited TNF-α-induced increase in ASMase activity and the release of ceramide. In addition, our study found that AMI promoted EC proliferation and viability and increased the phosphorylation of eNOS and endogenous NO production. Preincubation of EC with AMI prevented the downregulation of eNOS induced by TNF-α. These facts indicate the promising protective potential of AMI on ECs.

The original event in atherosclerosis is the accumulation of monocytes from the peripheral blood to the intima of the vascular wall [43]. Here, we found that AMI significantly inhibited the expression of adhesion molecules and decreased the secretion of sICAM-1 and sVCAM-1 in ECs activated by TNF-α. In addition, we found that AMI inhibited TNF-α-induced monocyte adhesion to endothelial cells. These results indicated that AMI prevents monocyte-endothelial adhesion by inhibiting the expression of TNF-α-mediated adhesion molecules. EC dysfunction has become a novel and important frontier in studies of cardiovascular disease prevention [31, 46–48]. Effective EC repair after injury is crucial to preventing atherosclerosis and vascular remodeling [49]. The present study found that TNF-α significantly inhibited EC functions, such as proliferation, migration, and tube formation in vitro, which was in line with a previous study [35]. Interestingly, AMI prevented TNF-α-induced endothelial dysfunction. Therefore, the above results indicate that AMI could reduce the damage to ECs induced by TNF-α, in turn enhancing the repair ability of ECs. ROS are crucial modulators of vascular tone, autophagy, and cytotoxicity and play an important role in the progression of cardiovascular disease [12, 50]. In vitro studies using ECs found that ASMase plays a role in TNF-α-induced mitochondrial ROS production [51]. A previous study also showed that inhibition of ASMase decreased the production of ROS derived from CER in RAECs induced by palmitic acid [52]. As expected, AMI significantly reduced TNF-α-induced production of ROS. Thus, AMI may improve impaired endothelial function by reducing ROS production.

Endothelial inflammation plays a crucial role in the progression of atherosclerosis. The MAPK/NF-κB signaling pathway is considered to be a key factor in regulating EC inflammation and plays a vital role in TNF-α-induced production of proinflammatory mediators in ECs [53]. Notably, NF-κB was also reported to regulate atherosclerotic lesion formation [54]. The expression of chemokines and adhesion molecules associated with endothelial inflammation in atherosclerosis has also been shown to be regulated by NF-κB [55]. The results revealed in this study suggested that in addition to the NF-κB pathway, the beneficial effects of AMI can be mediated by MAPK, which provides further insight into the mechanism.

Reduced ASMase was reported to induce vasodilation [56]; however, the role of AMI in endothelium-dependent relaxation under inflammation is not fully understood. In this study, we explored the function of AMI in mouse arterial dilation after intraperitoneal injection of recombinant mouse TNF-α. We noted that the endothelium-dependent dilation of the mouse aorta was impaired under the influence of TNF-α. Additionally, AMI and knockout of ASMase could enhance NO bioavailability and improve vasodilation in mice after injection of TNF-α. Consistent with the in vitro data, the immunofluorescence results indicated that ICAM was activated in the aortic endotheliocytes of inflammatory mice. Moreover, AMI and knockout of ASMase could significantly inhibit the level of ICAM, which further confirmed that AMI could ameliorate endothelial inflammation, in turn relieving vasodilation dysfunction.

To summarize, we discovered that preincubation with AMI ameliorated TNF-α-induced ASMase/CER and MAPK activation, which efficiently inhibited monocyte/EC interactions induced by TNF-α and reduced subsequent endothelial inflammation and endothelial dysfunction. Therefore, our results demonstrated that AMI has beneficial effects on endothelial dysfunction, which are mediated by its anti-inflammatory properties. These findings identify a novel role of AMI and provide a possible strategy for the prevention of vascular diseases.

### Supplementary Information

ESM 1 (DOCX 831 kb)

## Data Availability

The data supporting the findings of this study are available from the corresponding author upon request.
