# Supplementary material for: The Acid Sphingomyelinase Inhibitor Amitriptyline Ameliorates TNF-α-Induced Endothelial Dysfunction
Source: Cardiovasc Drugs Ther. 2022 Sep 14;38(1):43–56. doi: 10.1007/s10557-022-07378-0 (PMC10876840; doi:10.1007/s10557-022-07378-0)
Supplement: Supplementary file 1 — (DOCX 831 kb) [file 10557_2022_7378_MOESM1_ESM.docx]

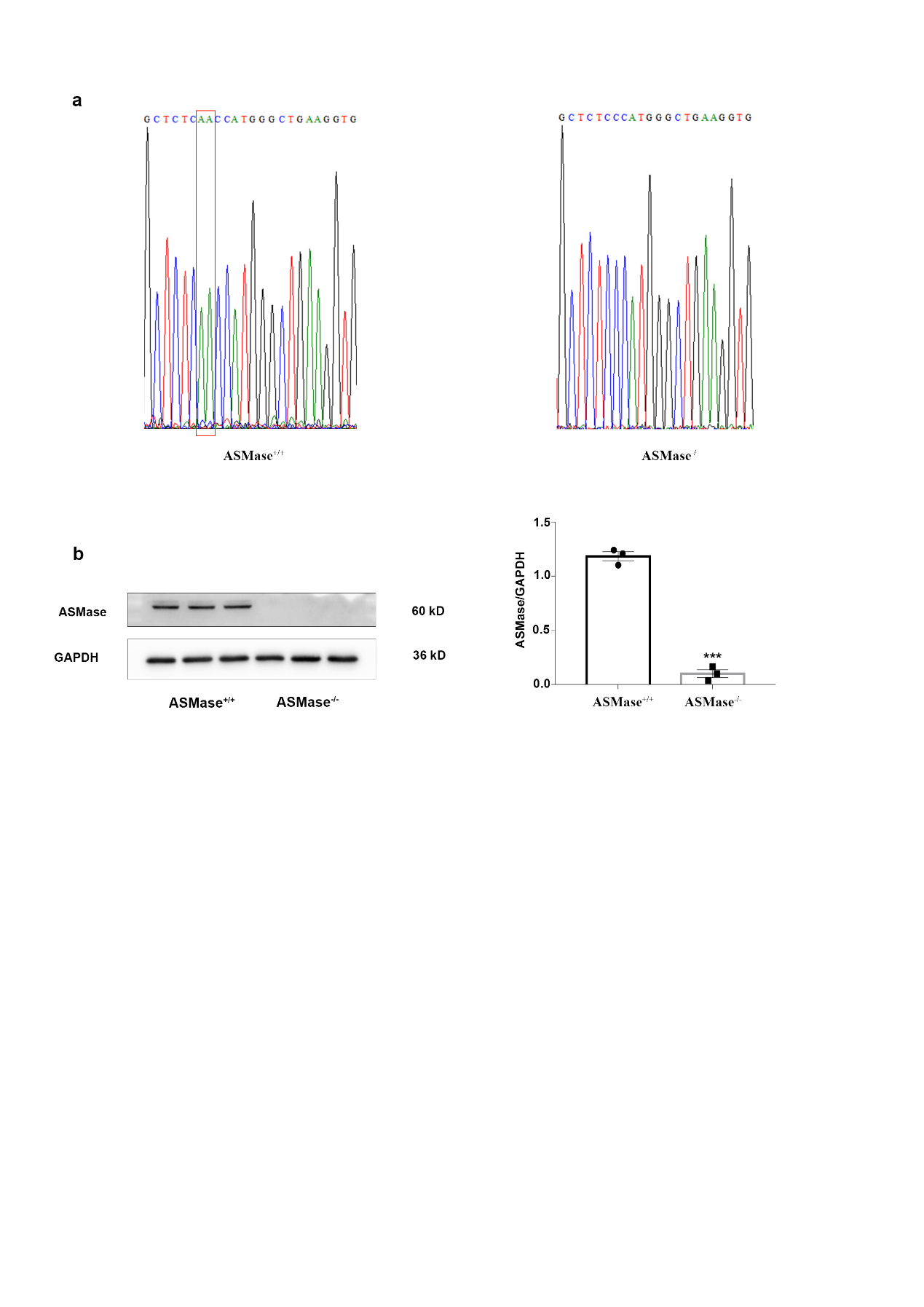


**Supplementary Fig.1 Identification of ASMase-/- mice** (a) Gene sequence diagrams of ASMase^+/+^ and ASMase^-/-^ mice. (b) Detection of ASMase protein in aorta tissues of ASMase^+/+^ and ASMase^-/-^ mice by western blotting. Data are represented as the mean±SEM; **P*<0.05, ***P*<0.01, ****P*<0.001 vs ASMase^+/+^.


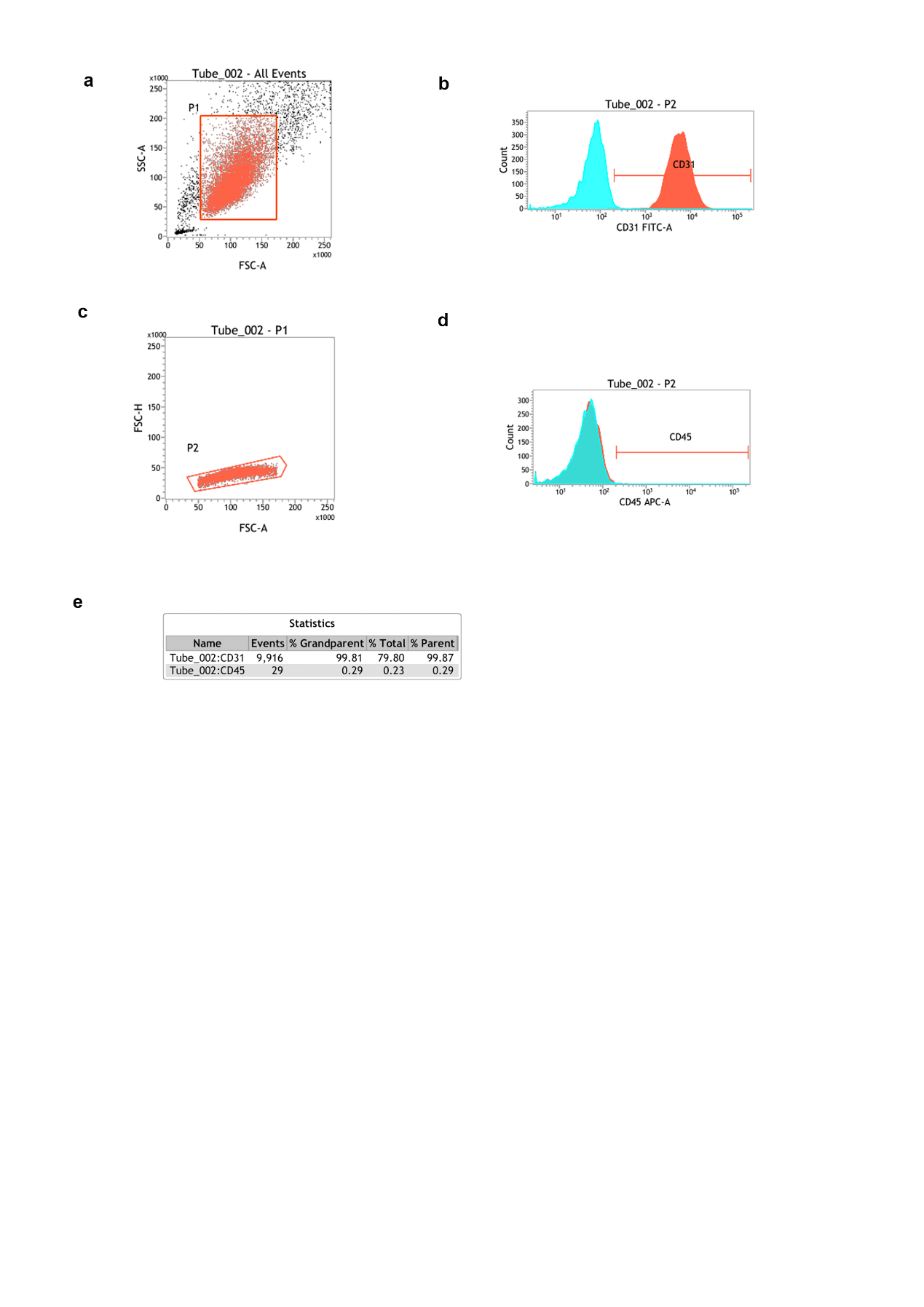


**Supplementary Fig.2 Identification of HUVEC** (a-d) The surface marker determination of HUVECs. (e) Statistics of CD31 and CD45 positive cells.


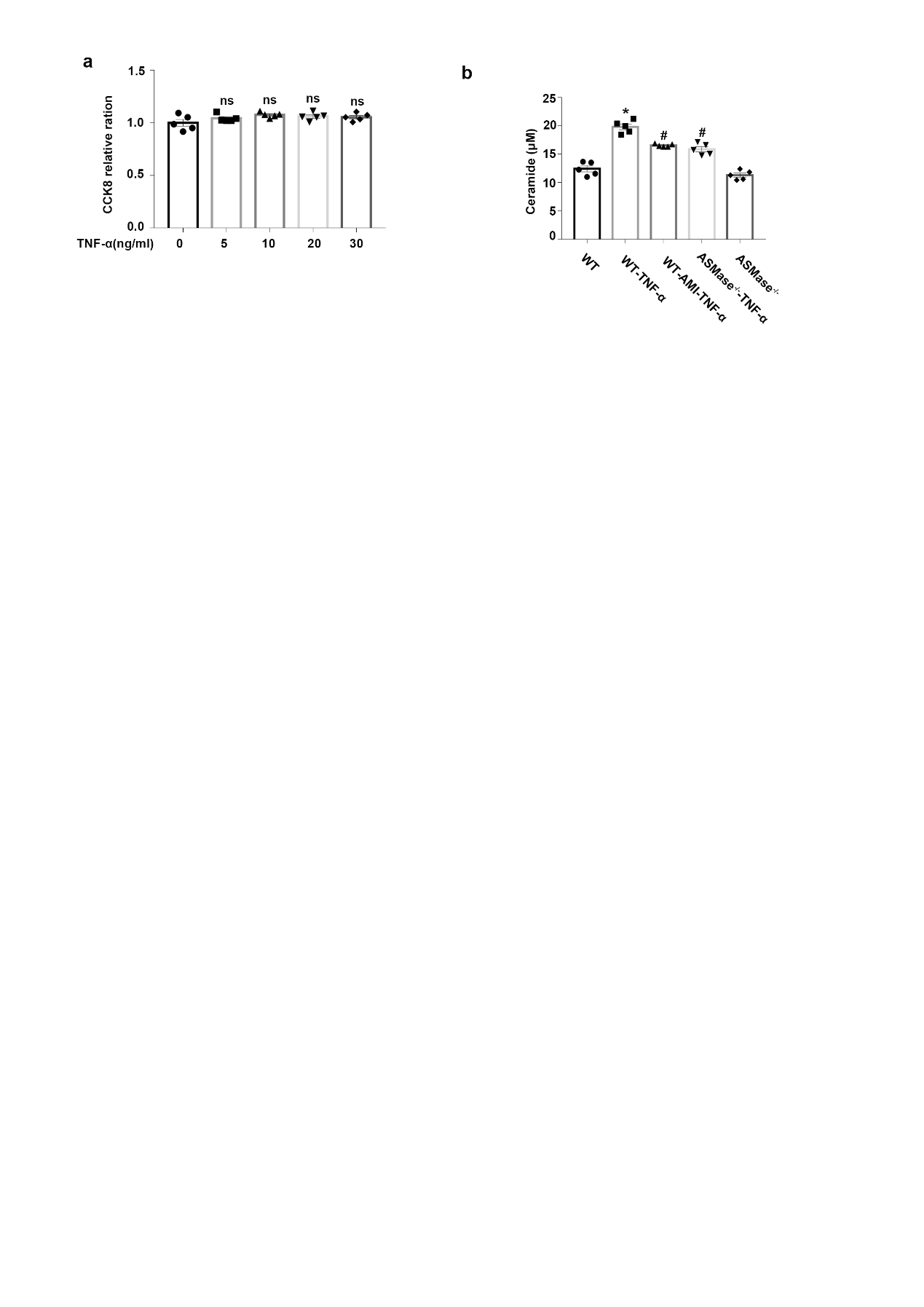


**Supplementary Fig.3** (a) Effects of TNF-α (0‐30 ng/ml, 24 hours) on the viability of HUVEC. (b) The concentration of ceramide in plasma of mice in all groups. Data are represented as the mean±SEM (n=3) **P*<0.05, ***P*<0.01, ****P*<0.001 vs control; #*P*<0.05, ##*P*<0.01, ###*P*<0.001 vs TNF-α, ns, no significance.
